# Glycosylphosphatidylinositol-anchored micronemal antigen (GAMA) interacts with the band 3 receptor to promote erythrocyte invasion by malaria parasites

**DOI:** 10.1016/j.jbc.2022.101765

**Published:** 2022-02-21

**Authors:** Jiachen Lu, Ruilin Chu, Yi Yin, Huijie Yu, Qinwen Xu, Bo Yang, Yifan Sun, Jing Song, Qiubo Wang, Jiahui Xu, Feng Lu, Yang Cheng

**Affiliations:** 1 Laboratory of Pathogen Infection and Immunity, Department of Public Health and Preventive Medicine, Wuxi School of Medicine, Jiangnan University, Wuxi, Jiangsu, China; 2 Department of Infectious Disease Control and Prevention, Shanghai Municipal Center for Disease Control and Prevention, Shanghai, China; 3 Department of Pathogen Biology and Immunology, School of Medicine, Yangzhou University, Yangzhou, Jiangsu, China; 4 Department of Gynecology, Affiliated Hospital of Jiangnan University, Wuxi, Jiangsu, China; 5 Department of Clinical Laboratory, Wuxi 9th Affiliated Hospital of Soochow University, Wuxi, Jiangsu, China

**Keywords:** *Plasmodium*, erythrocyte, membrane protein, receptor, invasion, microneme, GAMA, band 3, aa, amino acids, AMA1, apical membrane antigen 1, ANK1, ankyrin 1, EBA, erythrocyte-binding antigen, GFP, green fluorescent protein, His, histidine, IPTG, isopropyl β-d-1-thiogalactopyranoside, *K*_D_, equilibrium dissociation constant, MBD, membrane-binding domain, PfGAMA, *P. falciparum* glycosylphosphatidylinositol-anchored micronemal antigen, PI, preimmune, PvDBPII, *P. vivax* Duffy-binding protein region II, PvGAMA, *P. vivax* glycosylphosphatidylinositol-anchored micronemal antigen, RON2, rhoptry neck protein 2, SPR, surface plasmon resonance, Trx, thioredoxin

## Abstract

Glycosylphosphatidylinositol-anchored micronemal antigen (GAMA) is an erythrocyte binding protein known to be involved in malarial parasite invasion. Although anti-GAMA antibodies have been shown to block GAMA attachment to the erythrocyte surface and subsequently inhibit parasite invasion, little is known about the molecular mechanisms by which GAMA promotes the invasion process. In this study, LC-MS analysis was performed on the erythrocyte membrane to identify the specific receptor that interacts with GAMA. We found that ankyrin 1 and the band 3 membrane protein showed affinity for GAMA, and characterization of their binding specificity indicated that both *Plasmodium falciparum* and *Plasmodium vivax* GAMA bound to the same extracellular loop of band 3 (loop 5). In addition, we show the interaction between GAMA and band 3 was sensitive to chymotrypsin. Furthermore, antibodies against band 3 loop 5 were able to reduce the binding activity of GAMA to erythrocytes and inhibit the invasion of *P. falciparum* merozoites into human erythrocytes, whereas antibodies against *P. falciparum* GAMA (PfGAMA)-Tr3 only slightly reduced *P. falciparum* invasion. The identification and characterization of the erythrocyte GAMA receptor is a novel finding that identifies an essential mechanism of parasite invasion of host erythrocytes.

As apicomplexan parasites, malaria parasites infected 241 million people and caused 627,000 deaths worldwide in 2020 (1). Among the malaria parasite species that cause human infection, *Plasmodium falciparum* and *Plasmodium vivax* constitute the biggest threat to public health. *P. falciparum* leads to the greatest disease burden, while *P. vivax* is the most widespread human *Plasmodium* species. Although multiple intervention strategies have achieved different levels of success in controlling global malaria, the emergence of drug-resistant strains of *Plasmodium* and the absence of effective malaria vaccines are the major hurdles in malaria elimination (2, 3), mainly due to allelic polymorphism in *Plasmodium* populations and the lack of long-lasting immunologic memory (4, 5, 6). Other challenges are as follows: some antimalarial drugs could not be given to pregnant women, infants, and young children because of potential risks (7) and autoimmune and excessive inflammatory responses induced by vaccines may be detrimental to humans (8). Therefore, finding new approaches to vaccine and drug development for the treatment and prevention of malaria is essential.

All clinical symptoms and fatal malaria cases are largely attributed to asexual blood-stage infection. Thus, blockade of parasite invasion into erythrocytes could mitigate malaria disease. Specific interactions between multiple ligands on the parasite surface and their corresponding erythrocyte membrane receptors mediate complex erythrocyte invasion pathways. The interfaces of these interactions could be targets for vaccine and drug development (9). During parasite invasion, the proteins released from the apical secretory organelles, termed micronemes and rhoptries, may play an essential role, of which only several of their corresponding erythrocyte receptors have been identified (10). For instance, the interaction between *P. falciparum* reticulocyte binding homolog 5 (PfRh5)/PfRh5-interacting protein (PfRipr)/cysteine-rich protective antigen (CyRPA) complex and basigin is a separate and crucial step leading to *P. falciparum* invasion into erythrocytes (11, 12). Similarly, the interaction between *P. vivax* Duffy binding protein (PvDBP) and red blood cell Duffy antigen receptor for chemokines is involved in the formation of tight junction, resulting in successful invasion (13, 14).

In asexual blood stage, glycosylphosphatidylinositol-anchored micronemal antigen (GAMA) has been identified as a micronemal antigen of *Plasmodium* that binds to erythrocytes (15, 16). *Pfgama* could not be disrupted in *P. falciparum* strains 3D7 and W2mef, and immune responses to PfGAMA showed profound parasite-inhibitory effects, suggesting GAMA as a potential blood-stage vaccine candidate antigen (17). *P. vivax* infections induce robust IgG responses to *P. vivax* GAMA (PvGAMA) in natural exposure (18, 19), and anti-PvGAMA antibodies exhibit inhibitory activity on *Plasmodium knowlesi* (Pk) invasion and growth *in vitro*, although knockout of *pkgama* has a small effect on parasite growth (20). The function of host cell binding and invasion has also been found in *Toxoplasma gondii* GAMA (21). Similar with other microneme proteins, GAMA may be involved in the invasion process by interaction with specific erythrocyte membrane receptors.

In this study, to further understand the mechanisms underlying erythrocyte invasion by the malaria parasites *Plasmodium*, LC-MS was conducted to identify band 3 and ankyrin 1 (ANK1) as potential receptors for PfGAMA. By using additional biochemical interaction approaches, the fragment 2 of the membrane-binding domain (MBD) of ANK1 and extracellular loop 5 of band 3 was found to interact with GAMA in *P. falciparum* and *P. vivax*. Anti-band 3-P5 antibodies showed inhibitory activity on blocking GAMA binding to erythrocytes and *P. falciparum* invasion *in vitro*. The data provide direct mechanistic insight into malaria parasite invasion in blood stage, and a potential anti-band 3-P5 antibodies-based drug development strategy.

## Results

### Schematic of the primary structure of GAMA and expression and purification of the erythrocyte binding domain of GAMA

**Figure 1:**
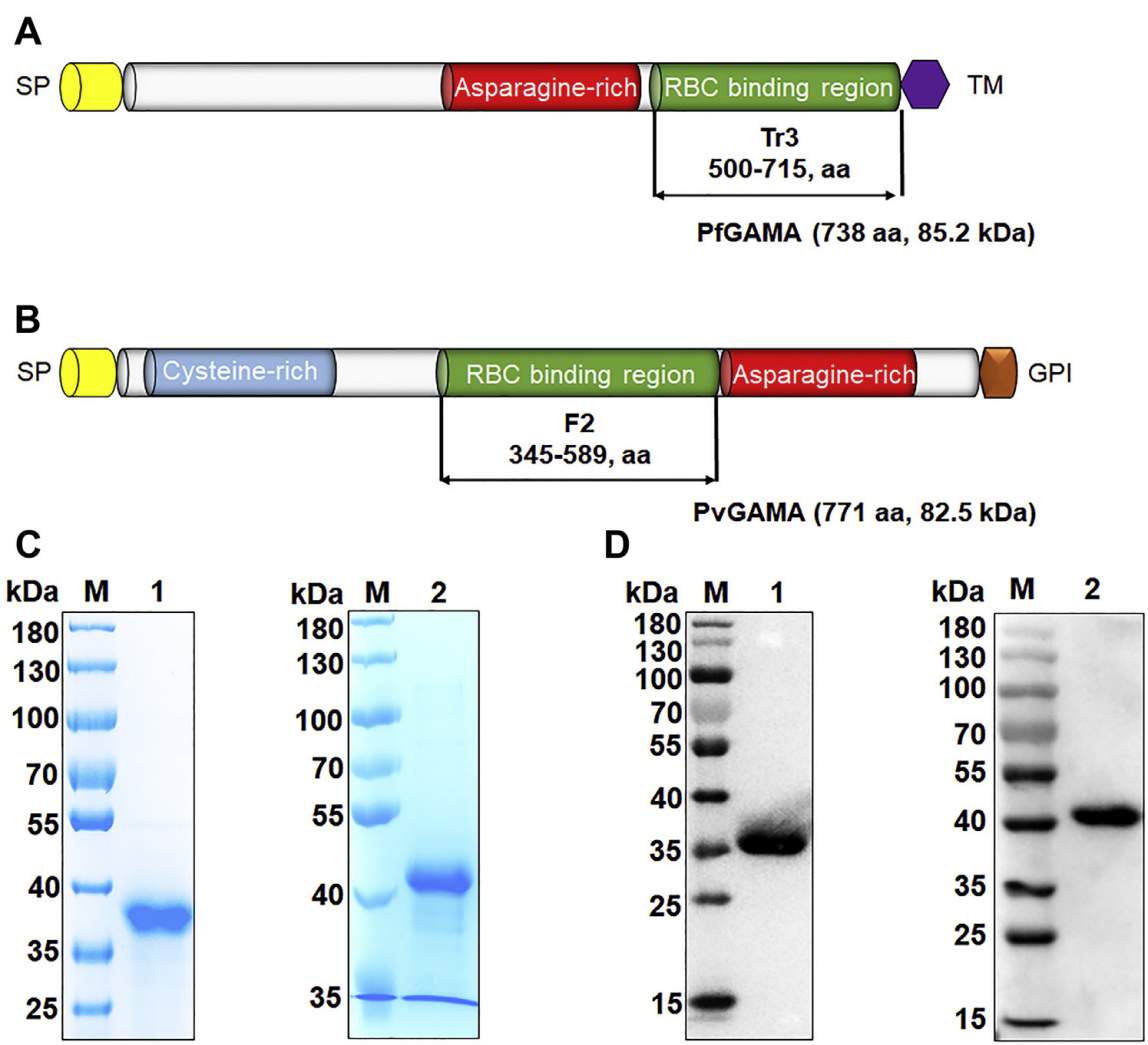
**Schematic diagram and expression of recombinant GAMA proteins.***A* and *B*, schematic representation of PfGAMA (*A*) and PvGAMA (*B*). Diagrams show the signal peptide (SP, *yellow*), cysteine-rich region (*blue*), asparagine-rich region (*red*), TM domain (*violet*), and GPI-anchor (*orange*). PfGAMA-Tr3 (aa 500–715) and PvGAMA-F2 (aa 345–589) were designed for cloning and expression. *C*, Coomassie blue-stained SDS-PAGE gels of purified recombinant proteins of GAMA. *D*, Western blot analysis of purified recombinant proteins of GAMA using an anti-His antibody. *Lanes M*, molecular mass marker. *Lanes 1*, PfGAMA-Tr3. *Lanes 2*, PvGAMA-F2. GAMA, glycosylphosphatidylinositol-anchored micronemal antigen; GPI, lycosylphosphatidylinositol; PfGAMA, P. falciparum glycosylphosphatidylinositol-anchored micronemal antigen; PvGAMA, P. vivax glycosylphosphatidylinositol-anchored micronemal antigen; TM, transmembrane.

The PfGAMA protein (encoded by PF3D7_0828800) is a 738-amino acid protein, that contains a signal peptide (amino acids [aa] 1–24), a long asparagine-rich region (aa 356–485), and a transmembrane domain (aa 715–738, Fig. 1*A*). The PvGAMA protein (encoded by PVX_088910) is a 771-amino acid protein, that contains an signal peptide (aa 1–21), an asparagine-rich region (aa 590–693), a cysteine-rich region (aa 54–220), and a glycosylphosphatidylinositol-anchor (aa 747–771, Fig. 1*B*). PfGAMA-Tr3 (aa 500–715) and PvGAMA-F2 (aa 345–589) were confirmed as the core erythrocyte binding region of PfGAMA and PvGAMA, respectively (15, 17). The codon-optimized *pfgama-tr3* and *pvgama-f2* genes, both with a Flag tag at the 3′ end, were cloned into the pET30a vector for histidine (His)-tagged recombinant protein expression in *Escherichia coli*. The soluble recombinant PfGAMA-Tr3 and PvGAMA-F2 proteins were purified with Ni-nitrilotriacetic acid-agarose to near homogeneity and migrated as a single band of approximately 36 and 41 kDa, respectively, under reducing conditions confirmed by SDS-PAGE and immunoblot using an anti-His antibody (Fig. 1, *C* and *D*).

### Identification of erythrocyte receptors for PfGAMA-Tr3 by affinity chromatograph and mass spectrometry

**Figure 2:**
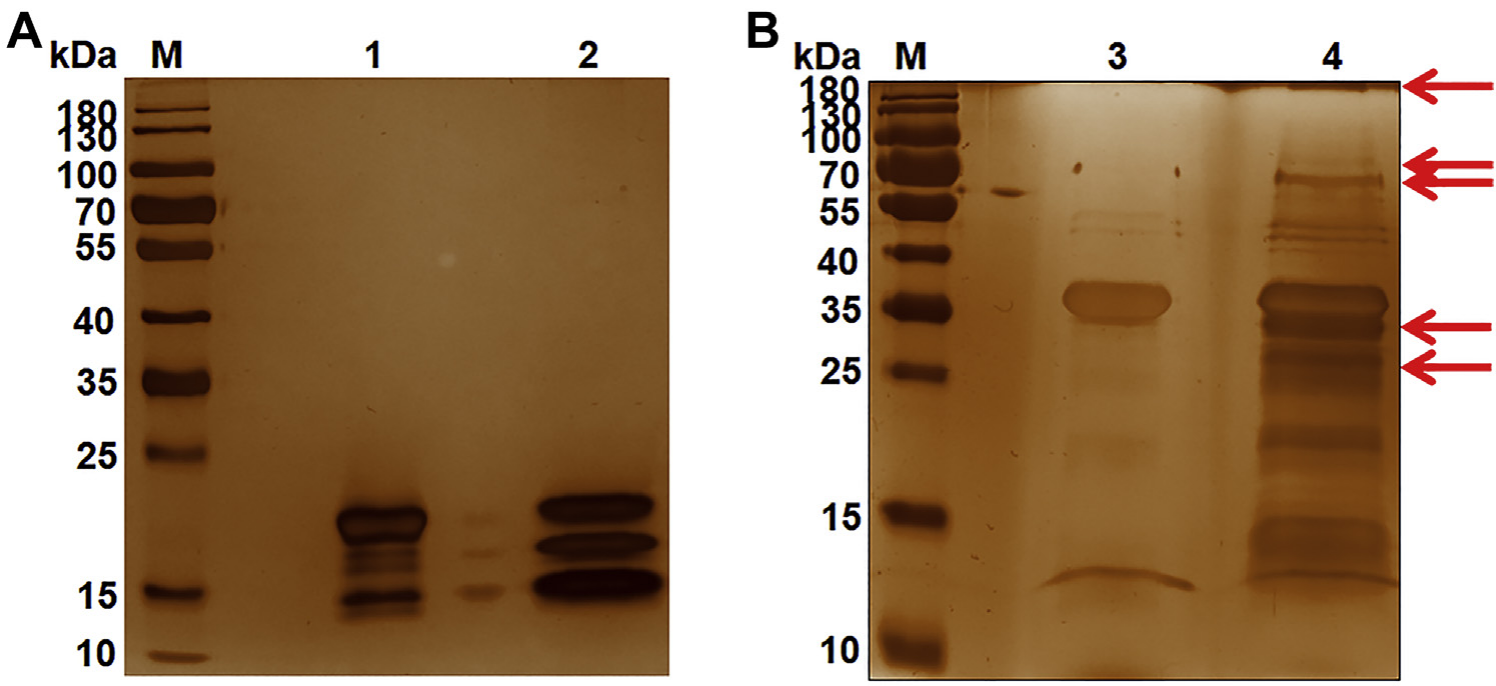
**Identification of erythrocyte membrane proteins binding to recombinant PfGAMA-Tr3 by co-affinity purification assay.***A* and *B*, erythrocyte membrane proteins were incubated with Trx-His tag protein (*A*) or recombinant His-tagged PfGAMA-Tr3 (*B*) bound to Ni-NTA resin, washed, eluted, and separated on silver-stained SDS-PAGE gels. *Lanes M*, molecular mass marker. *Lane 1*, Trx-His tag protein. *Lane 2*, erythrocyte membrane proteins incubated with Trx-His tag protein. *Lane 3*, PfGAMA-Tr3. *Lane 4*, erythrocyte membrane proteins incubated with PfGAMA-Tr3. *Arrowheads* indicate the differential protein bands. PfGAMA, *P. falciparum* glycosylphosphatidylinositol-anchored micronemal antigen.

**Figure 3:**
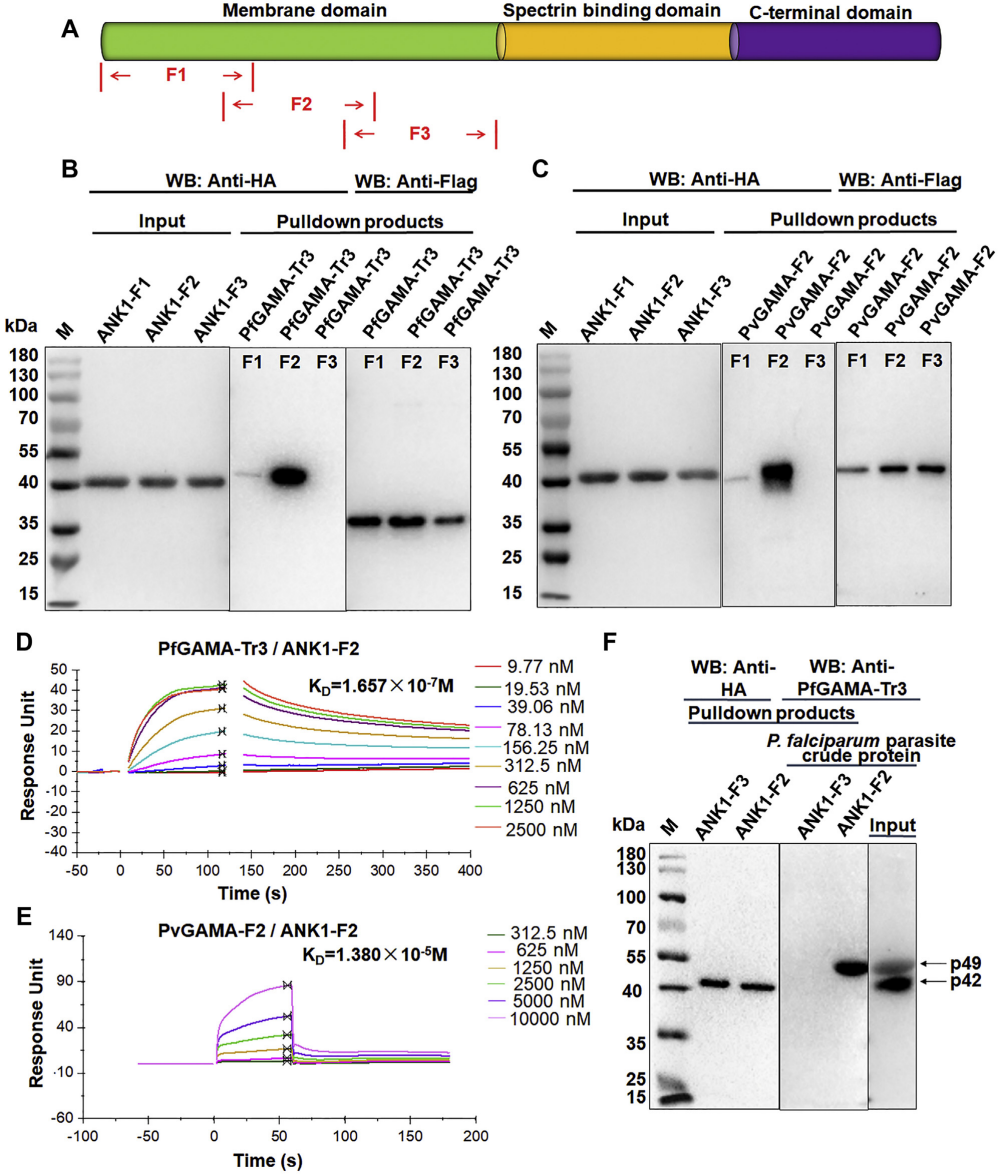
**Schematic diagram of ANK1 protein and interaction of GAMA with ANK1.***A*, schematic representation of ANK1 functional domains. The ANK1 protein consists of a membrane domain (*green*), a spectrin binding domain (*yellow*), and a C-terminal domain (*violet*). The membrane domain of ANK1 was divided into three (ANK1-F1, F2, F3) fragments for cloning, expression, and purification. *B* and *C*, Flag-pulldown assays showing that PfGAMA-Tr3 (*B*) and PvGAMA-F2 (*C*) bound to ANK1-F2 and weakly bound to ANK1-F1, but not to ANK1-F3. HA-ANK1-F1, F2, and F3 were incubated with immobilized Flag-PfGAMA-Tr3 or PvGAMA-F2, respectively. After washing the anti-Flag beads, the bound proteins were eluted and subjected to Western blot analysis using the indicated antibodies. *D* and *E*, SPR sensorgram for interaction between PfGAMA-Tr3 (*D*)/PvGAMA-F2 (*E*) and ANK1-F2. Recombinant PfGAMA-Tr3 (*D*) or PvGAMA-F2 (*E*) at indicated concentrations were injected over a CM5 chip onto which recombinant ANK1-F2 had been immobilized. The *K*_D_ value was determined by fitting a plot of response units at equilibrium against PfGAMA-Tr3 (*D*)/PvGAMA-F2 (*E*) concentrations. *F*, analysis of the binding of ANK1-F2 to native PfGAMA by HA-pulldown assays. *P. falciparum* parasite crude protein was incubated with immobilized ANK1-F3 (negative control) and ANK1-F2. The eluted proteins were probed by Western blot analysis using mouse anti-PfGAMA-Tr3 antisera. ANK1, ankyrin 1; PfGAMA, *P. falciparum* glycosylphosphatidylinositol-anchored micronemal antigen; PvGAMA, *P. vivax* glycosylphosphatidylinositol-anchored micronemal antigen.

To identify the erythrocyte receptors binding to PfGAMA-Tr3, we utilized the His-tagged PfGAMA-Tr3 protein immobilized to affinity resin as the bait protein to capture putative erythrocyte membrane proteins binding it. The purified and induced thioredoxin (Trx)-His tag protein of empty pET32a vector served as a negative control. After washing, the binding complexes were eluted using competitive analytes and separated on a silver-stained SDS-PAGE gel (Fig. 2). The differential protein bands were excised from the gels for in-gel digestion and LC-MS detection. Peptides that originated from seven kinds of erythrocyte membrane proteins were identified, including ANK1, band 3, protein 4.2, α-spectrin, protein 4.1, β-spectrin, and 55 kDa erythrocyte membrane protein (Table 1). Among them, ANK1 and band 3 had the two largest numbers of unique peptides identified. The ANK1 protein has three domains, comprising of a MBD (aa 1–827), a spectrin binding domain (aa 828–1382), and a C-terminal regulatory domain (aa 1383–1881) (22) as shown in Figure 3*A*. The MBD of erythrocyte ankyrin was a target for some *P. falciparum* exported proteins, such as PF3D7_0402000, *P. falciparum* histidine-rich protein 1, and knob-associated histidine-rich protein (23, 24, 25). Band 3 was also identified as an erythrocyte receptor for several *Plasmodium* invasion-related proteins, such as *P. falciparum* merozoite surface protein 1 (PfMSP1), *P. falciparum* merozoite surface protein 9 (PfMSP9), and *P. vivax* tryptophan-rich antigen 38 (PvTRAg38) (26, 27, 28). Collectively, the results indicated that ANK1 and band 3 are potential binding partners for GAMA.

**Table 1:** Identification of erythrocyte membrane proteins binding to PfGAMA-Tr3 in digested bands excised from the silver-stained gel by LC-MS

No.	Protein	UniProt accession no.	Coverage (%)	Molecular mass (kDa)	No. of unique peptides
1	Ankyrin 1	P16157	14.1	206.1	23
2	Anion transport protein (band 3)	P02730	18.8	101.7	15
3	α-Spectrin	P02549	3.2	280.0	7
4	Protein 4.2	P16452	9.6	77.0	6
5	Protein 4.1	P11171	6.9	97.0	6
6	β-Spectrin	P11277	1.8	246.5	4
7	55 kDa erythrocyte membrane protein	Q00013	2.2	52.3	1

Abbreviation: PfGAMA, *P. falciparum* glycosylphosphatidylinositol-anchored micronemal antigen.

### ANK1 interacts with PfGAMA and PvGAMA through its fragment 2

Other biochemical interaction analyses were carried out to validate whether ANK1 is an erythrocyte receptor for GAMA and map the core binding region of ANK1. First, the MBD of ANK1 was divided into three overlapping fragments, F1 (aa 1–318), F2 (aa 250–568), and F3 (aa 500–827, Fig. 3*A*). These three fragments were amplified using PCR to add HA-tag sequences at their 3′ end and cloned into pET30a for expressing as His-HA–tagged proteins. As shown by Western blotting, the observed molecular weights of ANK1-F1, -F2, and -F3 recombinant proteins were ∼42 kDa, consistent with the estimation (Fig. 3, *B* and *C*). Flag-pulldown assays were conducted to analyze the protein–protein interaction between GAMA and ANK1. PfGAMA-Tr3 or PvGAMA-F2 protein was immobilized on anti-Flag magnetic beads and incubated with ANK1-F1, -F2, and -F3 proteins. The results showed that PfGAMA-Tr3 and PvGAMA-F2 could bind to ANK1-F2 and weakly bind to ANK1-F1; however, no binding with ANK1-F3 was detected under the same conditions (Fig. 3, *B* and *C*). Additionally, the MBD (aa 1–827) of ANK1 was divided into four domains, D1 (aa 1–204), D2 (aa 205–402), D3 (aa 403–600), and D4 (aa 601–827) according to the previous study (29) for cloning, expression, and purification as above. The primers for PCR amplification were listed in Table S3. Flag-pulldown assays showed that PfGAMA-Tr3 and PvGAMA-F2 could bind to ANK1-D2 and weakly bind to ANK1-D1 (Fig. S1), indicating the rationality of the binding of GAMA to ANK1-F2. Thus, mapping analysis suggested that ANK1 interacted with PfGAMA and PvGAMA, mainly through the fragment 2 of MBD. Further, to provide additional evidence of interaction between PfGAMA and ANK1-F2, the binding affinity between them was quantitated by surface plasmon resonance (SPR) assays. PfGAMA-Tr3 or PvGAMA-F2 protein at varying concentrations was injected over a CM5 chip with immobilized ANK1-F2, and both were found to interact with ANK1-F2, with equilibrium dissociation constant (*K*_D_) values of 1.657 × 10^−7^ and 1.380 × 10^−5^ M, respectively (Fig. 3, *D* and *E*). To test whether ANK1-F2 protein recognizes native PfGAMA protein, HA pulldown assays were performed with *P. falciparum* parasite crude protein by using HA-tagged ANK1-F3 (negative control) and HA-tagged ANK1-F2 as baits. Consistent with the previous finding (17), anti-PfGAMA-Tr3 sera could detect p49 and p42 fragments in the *P. falciparum* parasite crude protein used as input. When ANK1-F2 was used as a bait, immunoblotting with mouse anti-PfGAMA-Tr3 sera revealed a strong band around 49 kDa (Fig. 3*F*), which was the size of the p49 fragment, a primary processing product of PfGAMA (16, 17). As expected, this band was not detected with the negative control.

### Extracellular loop 5 is the core region of band 3 bound to PfGAMA and PvGAMA

**Figure 4:**
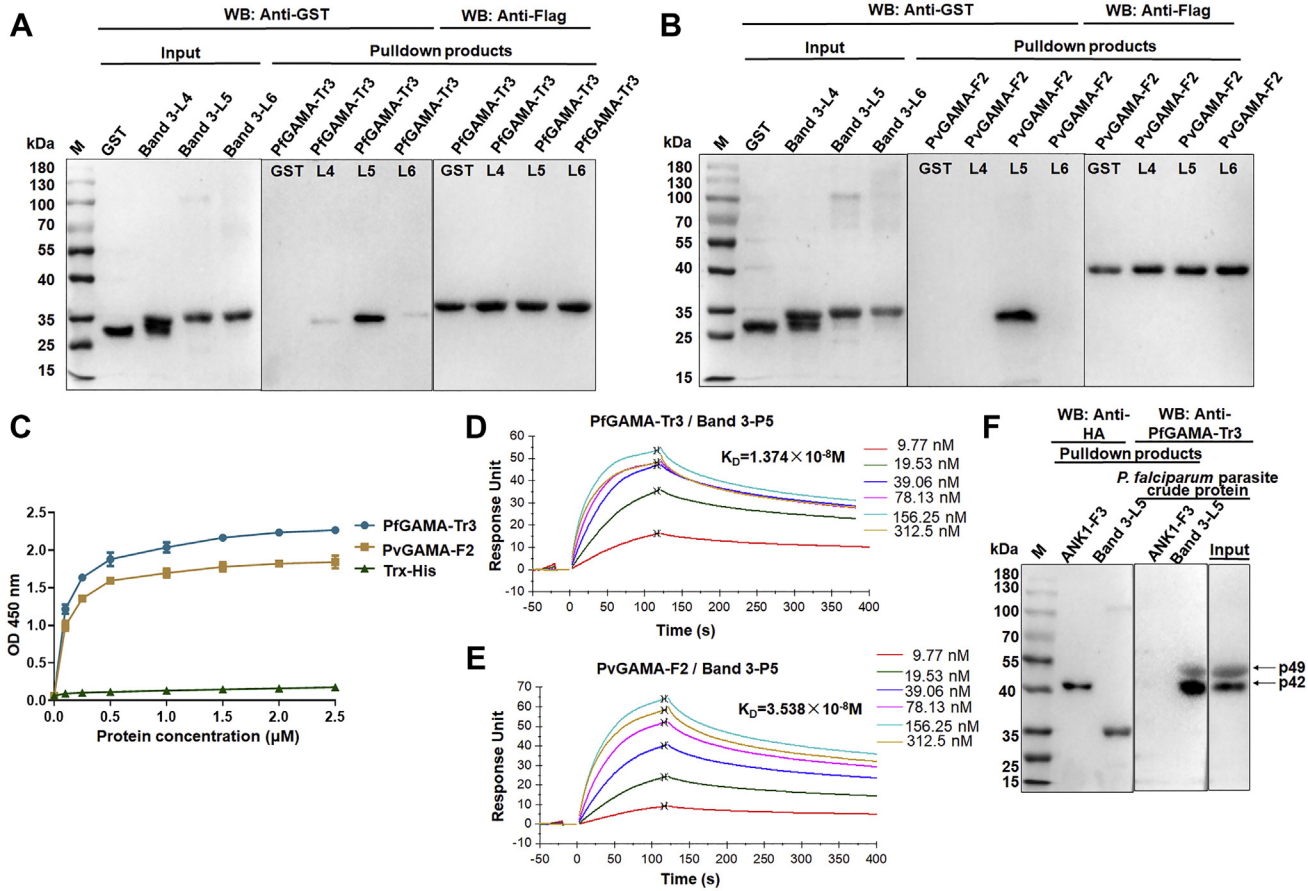
**Binding of GAMA to band 3.***A* and *B*, interaction of band 3 fragments with PfGAMA-Tr3 (*A*) and PvGAMA-F2 (*B*) analyzed by Flag-pulldown assays. GST and GST-tagged recombinant band 3 fragments (band 3-L4, L5, and L6) were incubated with immobilized Flag-PfGAMA-Tr3 (*A*) or PvGAMA-F2 (*B*), respectively. The eluted proteins were probed by Western blot analysis using anti-Flag and anti-GST antibodies. *C*, ELISA binding of PfGAMA-Tr3/PvGAMA-F2 to the plate wells coated with band 3-P5 peptide. Bound proteins were detected with a rabbit anti-GST antibody, followed by HRP-labeled goat anti-rabbit antibody. Trx-His tag protein was used as a negative control. Error bars represent SD (n = 3). *D* and *E*, SPR analysis showing the direct binding of PfGAMA-Tr3 (*D*)/PvGAMA-F2 © to band 3-L5 with high affinity. Synthetic band 3-P5 peptides (0–312.5 nM) were injected over the surface of immobilized PfGAMA-Tr3 (*D*) and PvGAMA-© (*E*). *F*, HA-pulldown assays showing recombinant band 3-L5 recognized native PfGAMA. Recombinant ANK1-F3 served as a negative control. ANK1, ankyrin 1; PfGAMA, *P. falciparum* glycosylphosphatidylinositol-anchored micronemal antigen; PvGAMA, *P. vivax* glycosylphosphatidylinositol-anchored micronemal antigen; SPR, surface plasmon resonance.

Similarly, a series of experiments was performed to confirm the binding of band 3 to GAMA. Thus, three of all six extracellular loops of band 3 (30), band 3-L4 (aa 623–663), band 3-L5 (aa 724–782), and band 3-L6 (807–857) were cloned, expressed, and purified with GST and HA tags on the basis of LC-MS identification results (Table S1). First, Flag-pulldown experiments were performed. As shown in Figure 4, *A* and *B*, strong interactions could be observed between GST-band 3-L5 and GAMA. Meanwhile, GST-band 3-L4 and GST-band 3-L6 were weakly bound to PfGAMA-Tr3, but not bound to PvGAMA-F2. These data indicated that extracellular loop 5 is the core region of band 3 bound to PfGAMA and PvGAMA. For further confirmation of this inference and to exclude reactivity with GST, ELISA assays were performed by incubating Trx-His tag protein (negative control) or recombinant His-tagged GAMA proteins with band 3-P5 peptide-coated ELISA plates. The results of ELISA showed that band 3-P5 bound to PfGAMA-Tr3/PvGAMA-F2 (Fig. 4*C*). SPR assay further verified the interaction between PfGAMA-Tr3/PvGAMA-F2 and band 3-P5 peptide, with *K*_D_ values of 1.374 × 10^−8^ and 3.538 × 10^−8^ M, respectively (Fig. 4, *D* and *E*). The p49 and p42 fragments of PfGAMA were pulled down by band 3-L5-immobilized beads from *P. falciparum* parasite crude protein, suggesting that band 3-L5 could also recognize native PfGAMA protein (Fig. 4*F*). Taken together, these results indicated that band 3 is another binding partner for PfGAMA and PvGAMA.

### Band 3 is a chymotrypsin-sensitive receptor for GAMA

**Figure 5:**
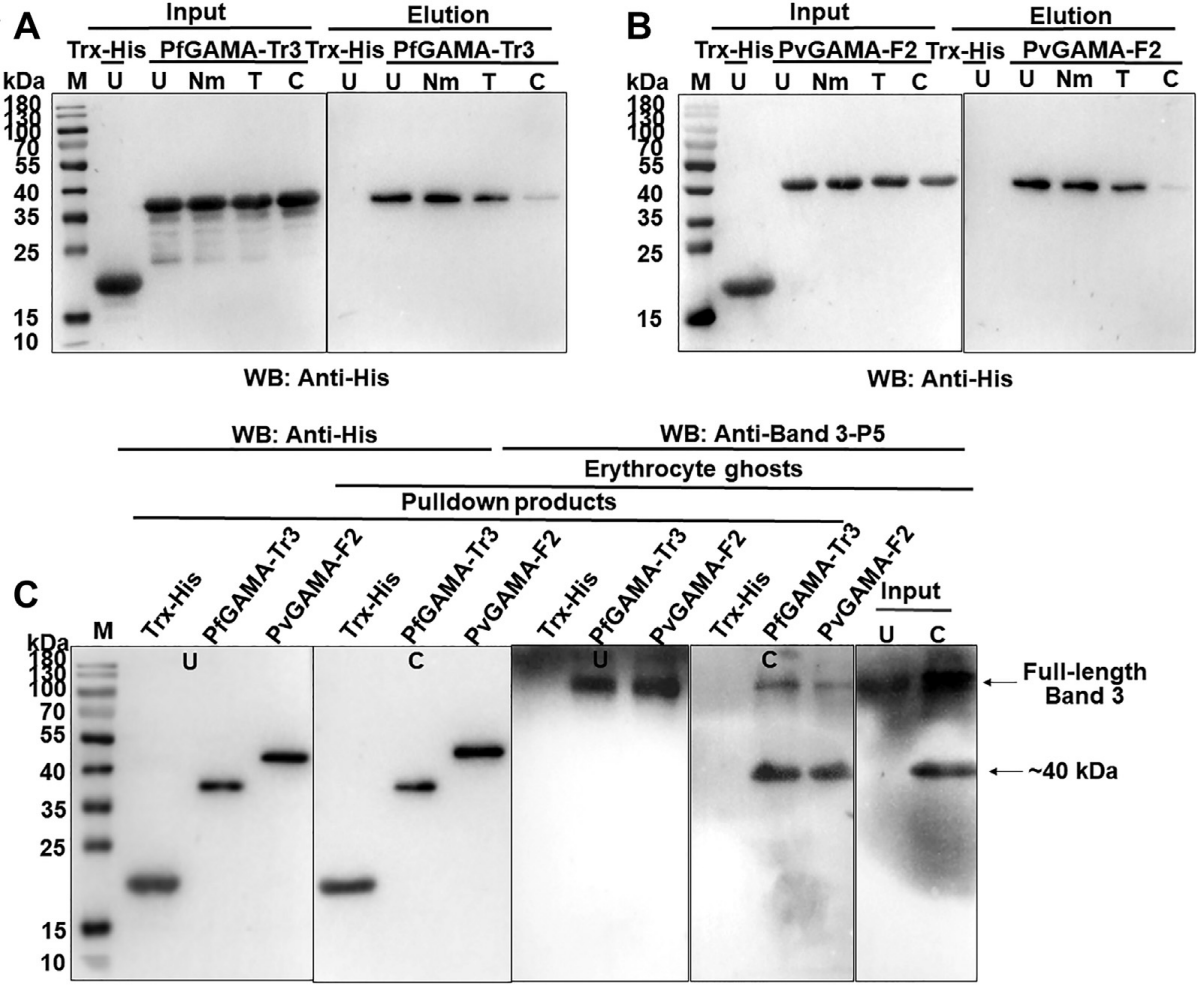
**Erythrocyte band 3 is a chymotrypsin-sensitive receptor for GAMA.***A* and *B*, receptor specificities for GAMA were analyzed by erythrocyte binding assay. PfGAMA-Tr3 (*A*) and PvGAMA-F2 (*B*) were incubated with untreated (U), neuraminidase-treated (Nm), trypsin-treated (T), and chymotrypsin-treated (C) erythrocytes to allow binding. Western blots showed GAMA binding was chymotrypsin sensitive. No binding was detected between Trx-His tag protein (negative control) and untreated erythrocytes. *C*, identification of the interaction between erythrocyte band 3 and GAMA using His-pulldown assays. Solubilized-untreated (U) and chymotrypsin-treated (C) erythrocyte ghosts were incubated with Trx-His tag protein, His-tagged PfGAMA-Tr3, or PvGAMA-F2 immobilized to the beads. Rabbit anti-band 3-P5 antisera were used for Western blot analysis to detect erythrocyte band 3 binding. PfGAMA-Tr3 and PvGAMA-F2 recognized full-length band 3 in both untreated and chymotrypsin-treated erythrocyte ghosts and specially recognized ∼40 kDa band in chymotrypsin-treated erythrocyte ghosts. PfGAMA, *P. falciparum* GAMA; PvGAMA, *P. vivax* GAMA.

Erythrocyte binding assays were performed using untreated or enzyme-treated erythrocytes to examine the erythrocyte receptor specificity of PfGAMA-Tr3 and PvGAMA-F2. As expected, Trx-His tag protein, a negative control, did not bind to untreated erythrocytes (Fig. 5, *A* and *B*). PfGAMA-Tr3 and PvGAMA-F2 bound to neuraminidase-treated erythrocytes with the similar binding affinity as untreated erythrocytes, whereas the binding of GAMA to erythrocytes was slightly reduced by treating erythrocytes with trypsin. It was worth noting that the treatment of erythrocytes with chymotrypsin substantially abolished the binding of GAMA to erythrocytes. These observations suggested that PfGAMA-Tr3 and PvGAMA-F2 bind to erythrocytes in a chymotrypsin-sensitive manner, replicating previous observations (15, 17). Previous studies have shown that erythrocyte band 3 is resistant to neuraminidase and trypsin but sensitive to chymotrypsin (26, 31). Additionally, we found that chymotrypsin treatment hardly affected erythrocyte ANK1 (Fig. S2). His-pulldown assays were performed by incubating solubilized untreated or chymotrypsin-treated erythrocyte ghosts with His-PfGAMA-Tr3 or His-PvGAMA-F2 protein immobilized on beads to further check whether band 3 is a chymotrypsin-sensitive receptor for GAMA. Antibodies raised against band 3-P5 detected full-length band 3 in solubilized ghosts, and they additionally detected a band of ∼40 kDa in chymotrypsin-treated ghosts (Fig. 5*C*). These bands were recognized in the products pull downed by GAMA-immobilized beads under these conditions (Fig. 5*C*). To sum up, these data suggested that PfGAMA-Tr3 and PvGAMA-F2 bind to human erythrocytes through the chymotrypsin-sensitive ∼40 kDa fragment of band 3.

### Antibodies against band 3-P5 inhibit PfGAMA and PvGAMA binding to erythrocytes

**Figure 6:**
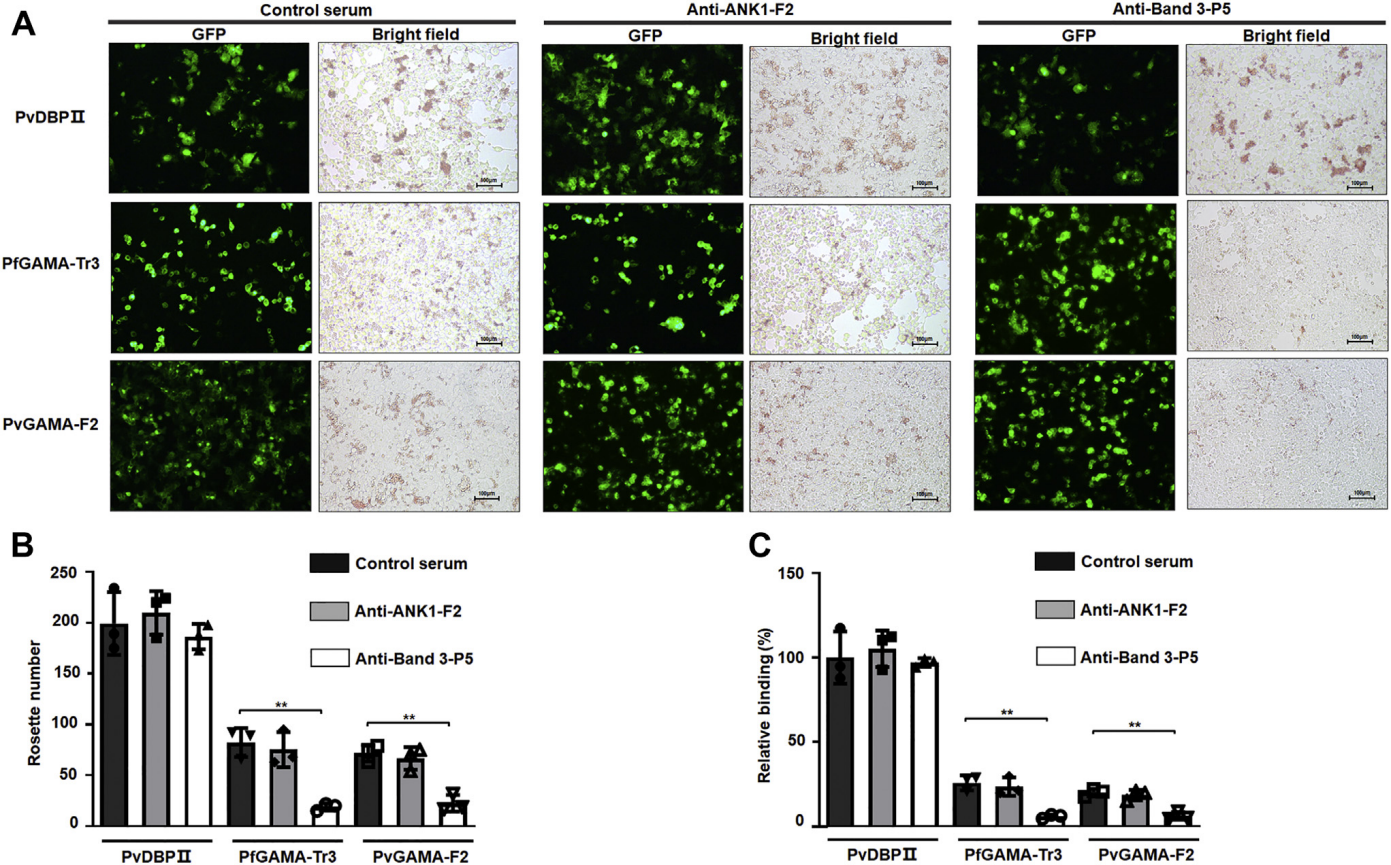
**Inhibition of erythrocyte binding to GAMA expressed on the surface of HEK293T cells by anti-ANK1-F2 antibodies or anti-band 3-P5 antibodies.***A*, HEK293T cell-based erythrocyte binding assay. PvDBPII, PfGAMA-Tr3, and PvGAMA-F2 were inserted into pEGFP-HSVgF1-N1 vector and transfected in HEK293T cells to be expressed on the surface of the cells. Erythrocytes were incubated with rabbit control serum (negative control) or immunized rabbit sera against ANK1-F2 or band 3-P5 diluted 1:100 in DMEM before addition. The transfection efficiency was evaluated by the proportion of GFP-positive cells using fluorescence microscopy, and the rosette formations were visualized under bright-field microscopy. The rosette formations were observed in PvDBPII-transfected or GAMA-transfected HEK293T cells when erythrocytes were treated with rabbit control serum or immunized rabbit sera against ANK1-F2 (bright-field images of left and middle panels). However, the rosette formations were seen in PvDBPII-transfected HEK293T cells, whereas few such formations were seen in GAMA-transfected HEK293T cells when treating erythrocytes with immunized rabbit sera against band 3-P5 (bright-field images of *right panels*). Scale bars, 100 μm. *B*, quantification of the rosettes formed by the transfected HEK293T cells expressing PvDBPII or GAMA when treating erythrocytes with rabbit control serum or immunized rabbit sera against ANK1-F2 or band 3-P5. *C*, inhibition of erythrocyte binding to PvDBPII or GAMA expressed on HEK293T cells by rabbit anti-ANK1-F2 antisera or rabbit anti-band 3-P5 antisera. Normalizing the rabbit control serum-treated erythrocyte binding capacity of PvDBPII (100% HEK293T cell transfection efficiency) as a standard (100%), the relative binding capacities under different conditions were calculated. Data are expressed as the mean ± SD of three separate experiments. Significant differences were determined using the Student’s *t* test. The *single asterisks*, *double asterisks*, and *triple asterisks* mark significant differences at the level of *p* < 0.05, *p* < 0.01, and *p* < 0.001, respectively. ANK1, ankyrin 1; DMEM, Dulbecco’s modified Eagle’s medium; PvDBP, *P. vivax* Duffy binding protein; PvDBPII, PvDBP region II; PvGAMA, *P. vivax* GAMA.

*In vitro* binding inhibition assay was performed to further investigate the inhibitory effect of rabbit antisera against ANK1-F2 or band 3-P5 on the PfGAMA-Tr3 and PvGAMA-F2 proteins binding to erythrocytes. PfGAMA-Tr3 and PvGAMA-F2 were expressed on the surface of HEK293T cells, and rosette-like structures formed by capturing multiple erythrocytes by transfected HEK293T cells were counted under bright-field microscopy (Fig. 6, *A* and *B*). Rabbit control serum served as a negative control. Meanwhile, PvDBP region II (PvDBPII) was introduced as a control to ensure the reliability and validity of the assay (32). The expression of PvDBPII, PfGAMA-Tr3, and PvGAMA-F2 on the surface of HEK293T cells was confirmed by the expression of green fluorescent protein (GFP). Transfection efficiency was evaluated by the proportion of GFP-positive cells under fluorescence microscopy (Fig. 6*A*, GFP). The transfection efficiencies of PvDBPII, PfGAMA-Tr3, and PvGAMA-F2 in HEK293T cells were approximately 25%, 40%, and 45%, respectively, in three independent assays. The binding rate equals rosette number/transfection efficiency/total cell number. The relative binding capacities under different conditions were calculated and normalized by setting the control serum-treated erythrocyte binding capacity of PvDBPII (100% HEK293T cell transfection efficiency) as a standard (100%). As shown in Figure 6*B*, the number of rosettes formed by GAMA-transfected HEK293T cells was significantly lower in the antibodies against band 3-P5-pretreated group than in the negative control group (*p* < 0.01). By contrast, no significant difference was found between these two groups with regard to the number of rosettes formed by PvDBPII-transfected HEK293T cells (Fig. 6*B*). Consistently, the antibodies against band 3-P5 reduced the binding activity of GAMA to erythrocytes, whereas they showed no inhibitory activity to block PvDBPII binding to erythrocytes (Fig. 6*C*). However, the antibodies against ANK1-F2 could not inhibit GAMA and PvDBPII binding to erythrocytes.

### Inhibition of *P. falciparum* invasion by antibodies against PfGAMA-Tr3, ANK1-F2, and band 3-P5

**Figure 7:**
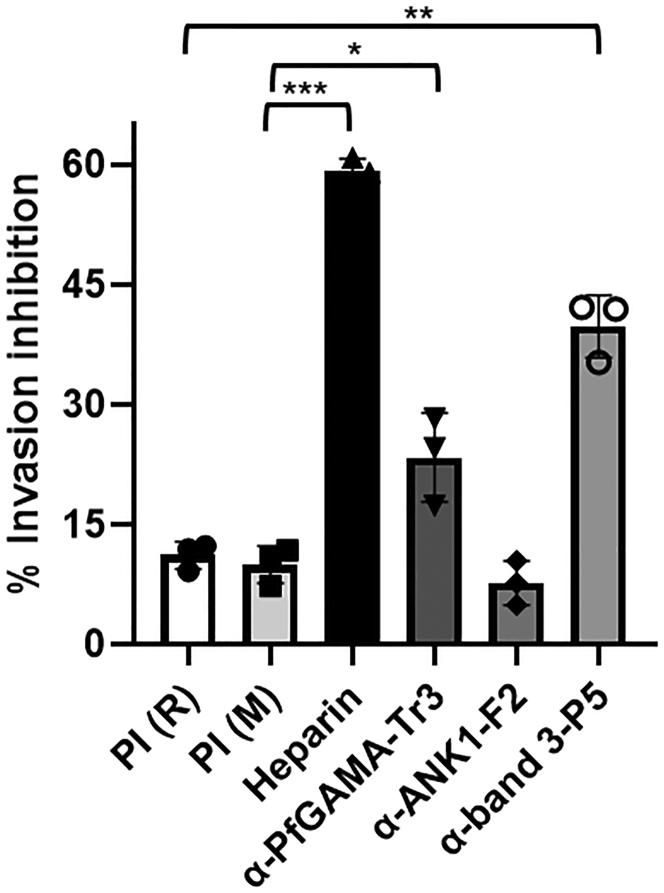
**Inhibition of *P. falciparum* invasion abilities by antisera.** Synchronized parasite cultures at the late trophozoite or early schizont stage were incubated with antisera, PI sera (negative control), and heparin (positive control). Live cells were gated based on forward scatter/sideways scatter characteristics, and parasitemia was determined by SYBR Green staining measured by flow cytometry for parasite cultures as compared with uninfected erythrocytes after 18 h. Error bars indicate mean ± SD, and statistical significance between PI sera and tested antisera was determined using one-way ANOVA with Tukey's post hoc test (∗*p* < 0.05, ∗∗*p* < 0.01 and ∗∗∗*p* < 0.001). *M*, mouse; PI, preimmune; *R*, rabbit.

The abilities of antibodies against PfGAMA-Tr3, ANK1-F2, and band 3-P5 to inhibit the invasion of *P. falciparum* into erythrocytes were tested through *in vitro* invasion assays in which heparin served as an invasion inhibition positive control. Invasion assays were performed by incubating synchronized parasite cultures with three tested antisera (anti-PfGAMA-Tr3, anti-ANK1-F2, and anti-band 3-P5), and infection rates were calculated by flow cytometry after 18 h under standard malaria culture conditions (Fig. S3). As shown in Figure 7, the rabbit anti-band 3-P5 sera at 1:10 dilution significantly inhibited erythrocyte invasion efficiency by 39.7% ± 3.9% compared with rabbit preimmune (PI) sera as a negative control, while mouse anti-PfGAMA-Tr3 sera showed weak inhibitory activity (22.3% ± 5.5%) compared with mouse PI sera. By contrast, rabbit anti-ANK1-F2 sera could not interfere with *P. falciparum* invasion into erythrocytes. Together, these data demonstrated that the antisera against band 3-P5 play an important part in blocking *P. falciparum* invasion into erythrocytes.

## Discussion

The invasion of erythrocytes by merozoites of *Plasmodium* is a complicated and multistep process involving quite a number of proteins (33). Elucidation of a series of events underlying invasion process and characterization of the molecular interactions involved are crucial for the development of antimalarial therapeutic interventions. GAMA has been identified and characterized as a novel *Plasmodium* erythrocyte binding protein and confirmed as a novel vaccine candidate. However, GAMA involved in machinery of host cell invasion was more attractive to the authors. Thus, in this study, band 3 was presented as the specific receptor of the erythrocyte membrane to GAMA. Moreover, anti-band 3-P5 antibodies could partially block GAMA attachment to the erythrocyte surface and inhibit *P. falciparum* invasion into erythrocytes. Together with the apparently conserved domain of GAMA–band 3 interaction, the finding suggested that this novel mechanism could provide an overall view of the invasion process of *P. falciparum* and *P. vivax* and a novel strategy for vaccine and drug development.

**Figure 8:**
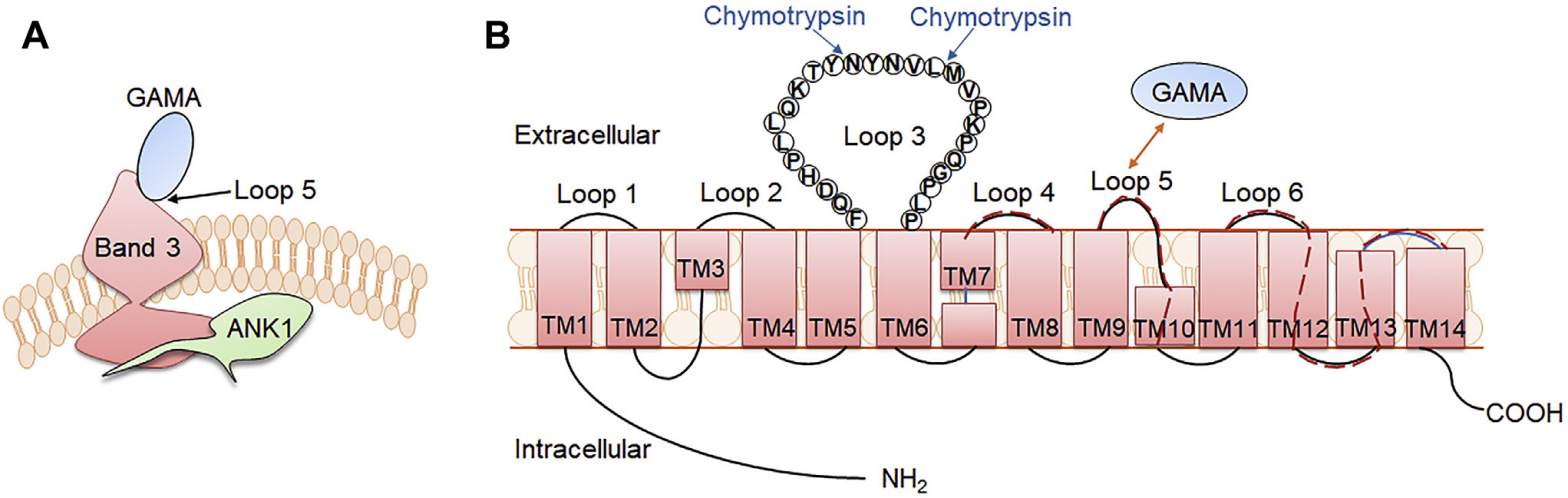
**Proposed possible models for GAMA binding to erythrocytes.***A*, GAMA directly binds to loop 5 of band 3, which is attached to the MBD of ANK1. *B*, PfGAMA recognizes a site within the chymotrypsin-sensitive C terminal ∼40-kDa fragment of band 3. This cartoon is drawn based on the recently published structure of human erythrocyte band 3 (64). *Red dashed lines* indicate the band 3 fragments (L4, aa 623–663; L5, aa 724–782; L6, aa 807–857) expressed in this study. The amino acid sequences of these three fragments are listed in Table S5. ANK1, ankyrin 1; MBD, membrane-binding domain; PfGAMA, *P. falciparum* glycosylphosphatidylinositol-anchored micronemal antigen; TM, transmembrane.

The micronemes among the apical organelles of merozoites were to be the first to secrete their protein contents, including erythrocyte-binding proteins, which are instrumental during erythrocyte invasion. *P. falciparum* erythrocyte-binding antigen (EBA)-175 is a microneme antigen with proven binding ability; it is expressed on the surface of transfected COS cells as a mediator of sialic acid-dependent binding (34). Glycophorin A (35) but not glycophorin B (36, 37) has been identified as an independent receptor for the EBA-175 binding pathway. Antibodies against PfEBA-175 have been shown to inhibit the binding of PfEBA-175 to erythrocytes and merozoite invasion in a concentration-dependent manner, suggesting that PfEBA-175 may be a malaria vaccine candidate (38, 39). Furthermore, the family of high molecular weight erythrocyte-binding protein microneme antigens has been found, including the DBP of *P. vivax* and *P. knowlesi* (40). GAMA was identified in *P. falciparum* and *P. vivax* to bind to erythrocytes; anti-PfGAMA antibodies inhibited merozoite invasion; and anti-PvGAMA antibodies inhibited erythrocyte binding, suggesting the adhesive role of GAMA. In the present study, ANK1 and band 3 were identified as potential receptors binding to PfGAMA or PvGAMA with affinity tags by using co-affinity purification plus mass spectrometry from erythrocyte membrane lysates (Table 1). ANK1 is the critical component of ankyrin complex, linking spectrin to band 3 and RhAG in the bilayer. ANK1 is divided into three structural domains, a N-terminal MBD, a central spectrin binding domain, and a C-terminal regulatory domain. MBD, which is folded into a nearly globular structure, consists of 24 copies of ∼33-aa tandem repeat (41, 42). ANK1 repeats interact with an assorted array of proteins, suggesting that they function as a general protein-binding motif (43). The MBD of ANK1 has also been confirmed as a receptor to *P. falciparum* knob-associated histidine-rich protein and another exported protein PF3D7_0402000 (23, 25). Band 3, also known as SLC4A1, which is attached to the MBD of ANK1, is the most abundant erythrocyte membrane protein that constitutes the core of membrane macrocomplex in the form of dimers or tetramers in the natural state (44). Based on these distinct characteristics, band 3 could serve as an adhesion receptor for a number of *Plasmodium* ligands to facilitate entry into erythrocytes (26, 27, 28, 45, 46). Meanwhile, antibodies against extracellular epitopes and the 5 C/6A regions of band 3 have been reported to exhibit an inhibitory effect on *P. falciparum* invasion (47, 48). Here, the fragment 2 (aa 250–568) involved in MBD and the extracellular loop 5 of band 3 that interacted with PfGAMA and PvGAMA were further mapped (Figs. 3 and 4). However, a question could arise that the absence of glycosylation site in the band3-L4 fragment expressed in this study might result in changes in its interaction with GAMA, due to the fact that loop 4 is glycosylated in the native protein, although removal of the N-glycosylation site through mutagenesis does not impair the functional expression of band 3 according to the previous reports (49, 50). Recombinant band 3-L5 could recognize the p49 and p42 fragments of native PfGAMA, while recombinant ANK1-F2 could only recognize p49 (Figs. 3*F* and 4*F*). p49 fragment is a primary processing product of full-length PfGAMA, resulting from a proteolytic cleavage event during transfer to the micronemes, and thus externalized to allow binding to erythrocytes (16). The previous studies have reported that processing of p49 gave rise to p42 and residual stub that was carried into the next erythrocyte upon invasion and remained associated with the ring-stage parasite (16, 17). Therefore, we speculate that *in vitro* recombinant ANK1-F2 selectively binds to PfGAMA at the site in the residual stub. Besides, the close apposition of the plasma membrane of the newly invaded parasite and the erythrocyte membrane was observed using indirect immunofluorescence assay with mouse anti-PfGAMA-Tr3 and rabbit anti-ANK1-F2 (Fig. S4). However, there is still no sufficient corroborating evidence for the interaction between the residual stub of GAMA and ANK1 *in vivo* in the current study. Additional experiments are needed to fully explore this interaction. In addition, anti-band 3-P5 antibodies could inhibit GAMA binding to the erythrocyte membrane, but not anti-ANK1-F2 antibodies, probably due to the fact that ANK1 faces the inside of the erythrocyte plasma membrane (Fig. 6). Meanwhile, this also points out that the co-affinity purification and LC-MS assays may have false positives, that is, ANK1 was pulled down by GAMA as a partner of band 3. The binding of GAMA to erythrocytes was sensitive to chymotrypsin treatment (Fig. 5, *A* and *B*), consistent with the published evidence in GAMA (15). Pulldown assays further showed that GAMA bound to human erythrocytes through the chymotrypsin-sensitive ∼40-kDa fragment of band 3 (Fig. 5*C*). On the basis of these findings, a possible model was proposed for GAMA binding to erythrocytes through band 3 under natural conditions (Fig. 8). GAMA directly attaches to a site within the chymotrypsin-sensitive C terminal ∼40-kDa fragment of band 3 which is bound to the MBD of ANK1. At present, our study does not provide direct evidence of the interaction between GAMA and ANK1. We will further study the stable interaction between GAMA, ANK1, and band 3 under natural conditions in the future work. In some cases, antibodies against parasite ligand alone could not be used effectively as antimalarial. Comparatively, antibodies against the host–parasite interaction complex or host receptor alone is more efficient. For instance, as a micronemal protein, apical membrane antigen 1 (AMA1), whose hydrophobic groove interacts with a conserved 49-aa loop of rhoptry neck protein 2 (RON2) in the invasion process (51, 52), is the leading blood-stage malaria vaccine candidate (53). The antibodies that bind near or in the hydrophobic groove of AMA1 interfered with *P. falciparum* invasion by blocking the binding to RON2 (54, 55). A vaccine composed of AMA1 in combination with RON2 may be more effective than AMA1 alone (56), suggesting a host–parasite-based approach toward developing a vaccine against malaria. Furthermore, PfRH5–basigin interaction is essential for merozoite invasion (12). Antibodies against basigin blocked erythrocyte invasion with no apparent toxicity, and they could be an efficacious treatment for patients with multidrug-resistant *P. falciparum* infection (57). In the present study, anti-PfGAMA-Tr3 antibodies, anti-ANK1-F2 antibodies, and anti-band 3-P5 antibodies were tested anti-invasion activity (Fig. 7). However, no evidence that anti-ANK1-F2 antibodies could interfere with *P. falciparum* invasion into erythrocytes was found. The possible reason is because ANK1 is not directly involved in the process of erythrocyte invasion by merozoites. A previous study reported that anti-full-length GAMA antibodies exhibited robust invasion-inhibitory activity (17), while another recent research showed that antibodies against PfMSP10 R1, which interacts with N-terminal PfGAMA, directly exhibited moderate invasion-inhibitory activity (58). However, here we observed that anti-PfGAMA-Tr3 sera did not inhibit erythrocyte invasion efficiency significantly. Thus, it is possible that a single truncation of PfGAMA alone could show low immunogenicity and efficacy, and it may also be due to that PfGAMA could form a complex with other merozoite proteins to act synergistically during the invasion process. Therefore, testing the antimalarial ability of antibodies raised against other truncations of GAMA and those against chimeric GAMA and other merozoite proteins in future studies is important. Although anti-PfGAMA-Tr3 antibodies exerted weak invasion-inhibitory activity, these results provide new insights into the merozoite invasion pathway involving GAMA. Moreover, as expected, anti-band 3-P5 antibodies partially blocked erythrocyte invasion. Further explorations are required to test the capability of anti-band 3-P5 antibodies for reducing *P. falciparum* invasion with a more rigorous evaluation using multiple *P. falciparum* isolates.

In conclusion, these data demonstrated the important role of GAMA–band 3 interaction in committing the merozoite for invasion. The results could help further understand the boundaries of the erythrocyte binding over the apical of the merozoite. Future work could be aimed at screening inhibitors of this interaction and evaluating the capability of naturally acquired antibodies to block GAMA binding or for protection efficiency.

## Experimental procedures

### Ethics statement

Human blood was obtained from healthy and consenting individuals into anticoagulant tubes containing EDTA, and the Medical Ethics Committee of Jiangnan University (JNU20190318IRB60) had approved the experiments involving human samples. All experiments involving animals were conducted according to approved protocol from the Animal Ethics Committee of Jiangnan University (JN. no. 20200530b0301031).

### Expression and purification of recombinant PfGAMA-Tr3 and PvGAMA-F2 proteins

The gene sequences of *pfgama* and *pvgama* were taken from PlasmoDB (http://plasmodb.org; accession no. PF3D7_0828800 and PVX_088910). The codon-optimized *pfgama-tr3* (aa 500–715) and *pvgama-f2* (aa 345–589) genes (Table S2) both with a Flag tag at the 3′ end were generated by DNA synthesis and cloned into *BamH*1/*Xho*1 sites of pET30a vector (Talen Biotech). *E. coli* BL21 (DE3) pLysS cells bearing these recombinant plasmids were grown in Luria–Bertani broth at 37 °C. The cells were induced at the optical density at 600 nm of 0.4 to 0.6 by adding 0.5 mM isopropyl β-d-1-thiogalactopyranoside (IPTG) and allowed to proceed for 7 h at 25 °C. Expression of Trx-His tag protein of empty pET32a vector was induced with 0.5 mM IPTG for 20 h at 16 °C. Soluble proteins were purified by Talen Biotech as described previously (59) with a slight modification using NPI10 (50 mM NaH_2_PO_4_, 300 mM NaCl, 10 mM imidazole, pH 8.0) as purification buffer. Additionally, the buffer of eluted proteins was exchanged to PBS using 10 kDa Amicon spin columns (Millipore).

### Co-affinity purification and LC-MS analysis

Purified His-PfGAMA-Tr3 and Trx-His tag protein (negative control) were used for the co-affinity purification of binding proteins from the erythrocyte membrane. First, the erythrocyte membrane was prepared according to Dodge *et al.* (60). Briefly, after washing the packed erythrocytes with cold PBS three times, a 40 volume of hypotonic lysis buffer (0.01 M Tris-HCl buffer, pH 7.4) was added and incubated with packed erythrocytes on ice for 20 min for hemolysis and then centrifuged at 10,000*g* for 30 min at 4 °C. The above procedures were repeated until erythrocyte ghosts appeared in the form of white pellets. The white pellets were lysed in five volumes of 1% C_12_E_8_ solubilizing buffer containing 1×protease inhibitor cocktail on ice for 20 min and were centrifuged to obtain supernatants (erythrocyte membrane proteins). His-PfGAMA-Tr3 or Trx-His tag protein (50 μg) was incubated with 100 μl equilibrated HisPur Ni-NTA Resin (Thermo Fisher Scientific) for 3 h at 4 °C with rotation, followed by washing three times with 200 μl wash buffer (20 mM NaH_2_PO_4_, 300 mM NaCl, 25 mM imidazole, pH 7.4). Erythrocyte membrane proteins (500 μg) were added and incubated with the resin bound to His-PfGAMA-Tr3 or Trx-His tag protein overnight at 4 °C. After washing the resin with 500 μl wash buffer to remove the unbound proteins, the bound complexes were eluted using 200 μl elution buffer (20 mM NaH_2_PO_4_, 300 mM NaCl, 250 mM imidazole, pH 7.4). The eluted proteins and purified His-tagged proteins were mixed with 5 × SDS reducing loading buffer, boiled and loaded on SDS-PAGE. The gels were then stained using the fast silver stain kit (Beyotime Biotechnology).

The differential protein bands were excised from the gels and digested using in-gel tryptic digestion method according to a published protocol (61). Digests were transferred to a fresh EP tube, and remaining peptides were extracted from the gel with 60% acetonitrile containing 0.1% trifluoroacetic acid followed by ultrasonic treatment. The extracts were combined, dried in a vacuum concentrator, and were dissolved in 0.1% formic acid for LC-MS analysis with a Q-Exactive mass spectrometer (Thermo Fisher Scientific) coupled to an Easy-nLC 1000 instrument (Thermo Fisher Scientific). The peptides were loaded into an Acclaim PepMap RSLC C18 column (100 μm × 2 cm, Thermo Fisher Scientific) and separated in an EASY column (10 cm, ID75 μm, 3 μm, C18-A2, Thermo Fisher Scientific) with a 30-min linear gradient at a flow rate of 300 nl/min. The eluted peptides were directly injected into the electrospray ion source and detected in the scanning range of 300 to 1800 *m/z* with the resolution for MS1 and MS2 *r* = 70,000 and 17,500 at *m/z* 200. Twenty fragments (MS2 scan) were acquired following each full scan. Peaklists generation and database search were performed using Proteome Discoverer 1.4 (Thermo Fisher Scientific) by Shanghai Applied Protein Technology Co, Ltd. Peptides and proteins were identified using a UniProt database of *Homo sapiens* (downloaded on 17 February 2020) containing 188,433 entries. The search parameters were as follows: precursor ion mass tolerance as 20 ppm, fragment ion mass tolerance as 0.1 Da, and trypsin digestion allowing one missed cleavages. Carbamidomethylation of cysteine and methionine oxidation were specified as fixed modification and variable modification, respectively. The processed data were filtered in the Proteome Discoverer 1.4 software with the peptide validator algorithm (q-value < 0.01) to ensure the peptide spectrum match had a false discovery rate under 1%. The expected value was adjusted to match the false discovery rate of 1%.

### Cloning, expression, and purification of recombinant ANK1 proteins and band 3 proteins

To generate HA-tagged fusion proteins, each DNA fragment encoding ANK1-F1 (aa 1–318), ANK1-F2 (aa 250–568), ANK1-F3 (aa 500–827), or the last three extracellular loops of band 3 (band 3-L4, L5, L6) containing a HA tag at the C terminal was amplified from the cDNA (reversely transcribed by the human reticulocyte mRNA) using six respective reverse primers which introduced the HA tag nucleotide sequence and six respective forward primers listed in Table S3. PCR products were cloned into the *BamH*I and *Xho*I sites of pET30a or pGEX-6P-1 vector. The pET30a plasmids carrying ANK1-F1, -F2, -F3 gene fragments and the pGEX-6P-1 plasmids carrying band 3-L4, L5, L6 gene fragments were transformed into *E. coli* BL21 (DE3) pLysS cells for expression. Protein expression was induced with 0.5 mM IPTG for 7 h at 37 °C. Soluble proteins were purified by Talen Biotech as described previously (62). Additionally, the buffer of eluted proteins was exchanged to PBS using 10 kDa Amicon spin columns (Millipore).

### Production of antisera raised against PfGAMA-Tr3, ANK1-F2 and band 3-P5

To generate mouse anti-PfGAMA-Tr3 antisera, five female BALB/c mice (6–8 weeks old) were immunized intraperitoneally with 50 μg of PfGAMA-Tr3 protein mixed in Freund’s complete adjuvant (Sigma), followed by two intraperitoneal injections with the same amount of PfGAMA-Tr3 mixed in Freund’s incomplete adjuvant (Sigma) for boosting at 3-weeks intervals. To avoid immune response against GST, band 3-P5 peptide was synthesized (Table S4). To generate rabbit anti-ANK1-F2 antisera and rabbit anti-band 3-P5 antisera, one female New Zealand white rabbit (2 months old) was injected intramuscularly with 500 μg of ANK1-F2 protein or BSA-coupled band 3-P5 peptide mixed in Freund’s complete adjuvant, followed by two boost injections with 500 μg of ANK1-F2 or BSA-coupled band 3-P5 mixed in Freund’s incomplete adjuvant at 4-weeks intervals. The prime and the final boosts were given by intramuscular injection and intravenous injection, respectively. Antisera were collected 2 weeks after the final boost. Among the above experiments, production of rabbit antisera was carried out by YouLong Biotech.

### Pulldown assays

To evaluate the interaction between GAMA and ANK1/band 3 and identify the core binding region of ANK1 or band 3, Flag-pulldown assays were carried out. PfGAMA-Tr3 or PvGAMA-F2 protein (12 μg) were incubated with 20 μl anti-Flag M2 magnetic beads (Sigma) for 2 h at 4 °C to immobilize on beads. The beads were collected using a magnetic separator and incubated separately with purified HA-ANK1-F1, F2, F3, GST, GST-band 3-L4, L5, and L6 proteins for 3 h at 4 °C. After washing the beads with 20 volumes of TBS buffer (50 mM Tris HCl, 150 mM NaCl, pH 7.4) three times, the beads were resuspended in 50 μl of 1 × SDS reducing loading buffer and boiled for 8 min. Supernatant was used for Western blot analysis.

To test whether ANK1-F2 and band 3-L5 proteins recognize native PfGAMA protein, anti-HA magnetic beads (Bimake) containing approximately 20 μg of the indicated HA-tagged proteins were incubated with *P. falciparum* parasite crude protein at 4 °C overnight. After washing with 500 μl PBS with 0.5% Tween-20 for five times, the beads were boiled, and Western blot analysis was conducted as above.

To detect native band 3 binding of recombinant GAMA, His-pulldown assays were performed by incubating anti-His-tag mAb-magnetic agarose (MBL) containing 30 μg of Trx-His-tag protein or His-tagged GAMA proteins with 300 μg of solubilized untreated or chymotrypsin-treated erythrocyte ghosts for 3 h at 4 °C with gentle agitation. After washing with TBS buffer, the beads were boiled, and Western blot analysis was conducted as above.

### SDS-PAGE and Western blot analysis

Purified recombinant proteins were mixed with 5 × SDS reducing loading buffer and boiled for 8 min at 100 °C and then resolved on 10% to 12% SDS-PAGE followed by Coomassie brilliant blue staining. For Western blot analysis, purified recombinant proteins, protein pulldowns, and eluted proteins were separated by 10% to 12% SDS-PAGE and transferred onto polyvinylidene difluoride membranes (Millipore) and block with 5% skimmed milk in Tris-buffered saline-Tween 20 for 2 h at room temperature (RT). After washing with Tris-buffered saline-Tween 20 three times, the polyvinylidene difluoride membranes were incubated with primary antibodies overnight at 4 °C and then with a horseradish peroxidase-labeled goat anti-mouse or anti-rabbit IgG antibody (SouthernBiotech) for 1.5 h at RT. The fluorescence signals were visualized using an enhanced chemiluminescence kit (New cell & Molecular Biotech) and analyzed using a ChemiDoc MP imaging system (Bio-Rad). The following primary antibodies were used: mouse anti-His antibody (ABclonal), mouse anti-HA antibody (ABclonal), rabbit anti-GST antibody (Proteintech), rabbit anti-Flag antibody (Proteintech), mouse anti-PfGAMA-Tr3 antisera, rabbit anti-ANK1-F2 antisera, and rabbit anti-band 3-P5 antisera.

### ELISA binding of PfGAMA-Tr3/PvGAMA-F2 to band 3-P5

To exclude reactivity with GST and check whether recombinant GAMA binds to band 3-L5, ELISA assays were performed. Briefly, 96-well ELISA plates were coated with 100 ng of band 3-P5 peptide (1 μg/ml) dissolved in carbonate/bicarbonate buffer per well at 4 °C overnight and then blocked with 0.5% BSA in PBS containing 0.05% Tween-20 (PBST) for 2 h at RT. The plates were washed with PBST three times and incubated with recombinant PfGAMA-Tr3/PvGAMA-F2 protein or Trx-His tag protein at different concentrations for 2 h at RT. After washing again, bound proteins were detected with rabbit anti-GST antibody, followed by horseradish peroxidase-labeled goat anti-rabbit antibody. Subsequently, the plates were washed and developed with TMB substrates (Invitrogen). After stopping the reaction by adding 50 μl of 2 M H_2_SO_4_ per well, the optical density was measured at 450 nm.

### SPR analysis

To determine the affinity of the binding of GAMA to ANK1-F2/band 3-L5, SPR analyses were conducted on a BIAcore S200 instrument (GE Healthcare) at 25 °C using HBS-EP (10 mM Hepes, 150 mM NaCl, 3 mM EDTA, and 0.005% v/v Surfactant P20, pH 7.4) as running buffer. Flow cell 1 of a CM5 sensor chip (GE Healthcare) without any ligand served as a reference to remove the nonspecific binding. Recombinant ANK1-F2 or band 3-P5 peptide in 10 mM NaAC, pH 4.5, was immobilized in another flow cell at a flow rate of 10 μl/min. The analytes, varying concentrations of recombinant PfGAMA-Tr3 or PvGAMA-F2, were flowed over both immobilized ANK1-F2/band 3-P5 and flow cell 1 at a flow rate of 30 μl/min for 2 min followed by 5-min dissociation. The CM5 sensor chip was regenerated with 15 μl 10 mM glycine-HCl, pH 2.0. For kinetics analysis, response units at steady state were plotted against analyte concentrations and fitted to a 1:1 Langmuir binding model using Biacore S200 evaluation software (GE Healthcare). The value of *K*_D_ was calculated as *k*_d_/*k*_a_, where *k*_d_ and *k*_a_ are the dissociation and association rate constants, respectively.

### Enzymatic treatment of erythrocytes and binding assays

The protocol for the enzymatic treatment of erythrocytes was as published previously (17, 26). Two hundred fifty microliter of packed erythrocytes were incubated with neuraminidase (66.7 mU/ml), trypsin (1 mg/ml), or chymotrypsin (1 mg/ml) in 1-mL final volume of incomplete RPMI medium for 1 h at 37 °C with constant rocking. The trypsin or chymotrypsin-treated samples were further incubated with a trypsin inhibitor (0.5 mg/ml) for 10 min at 37 °C to inactivate the enzymes. The erythrocytes were extensively washed with incomplete RPMI 1640 medium, followed by the binding assay.

To perform the erythrocyte binding assay of recombinant GAMA, 100 μl of untreated or enzyme-treated packed erythrocytes were incubated with 10 μg of Trx-His tag protein, His-PfGAMA-Tr3, or His-PvGAMA-F2 protein for 1 h at RT to allow binding. The pelleted erythrocytes were collected by centrifugation through 300 μl silicon oil. After washing the erythrocytes with PBS once, bound proteins were eluted using PBS containing 0.5 M NaCl.

### HEK293T cell-based erythrocyte binding inhibition assay by rosetting

This assay was performed as noted earlier with slight modifications (31). For expression on the surface of HEK293T cells, *pfgama-tr3* and *pvgama-f2* were amplified from pET30a-PfGAMA-Tr3 and pET30a-PfGAMA-F2 plasmid DNA, respectively, using In-fusion primers listed in Table S3, and cloned into the *EcoR*I and *Apa*I sites of pEGFP-HSVgD1-N1 vector. These constructs were purified using a PurePlasmid Mini Kit (CWBIO Biotech).

HEK293T cells (1.5 × 10^5^/well) were cultured in 24-well plates and transfected with each of the above recombinant DNAs (500 ng/well) using Lipofectamine LTX & PLUS Reagent (Invitrogen) in sera-free Dulbecco’s modified Eagle’s medium followed by incubation in a humidified incubator (5% CO_2_, 37 °C) for 30 h. Meanwhile, HEK293T cells transfected with plasmid PvDBPII (31) served as a control to ensure the reliability and validity of the assay. Human erythrocytes (1% hematocrit) were preincubated with rabbit control serum or immunized rabbit sera against ANK1-F2 or band 3-P5 (diluted 1:100 in incomplete Dulbecco’s modified Eagle’s medium) at 37 °C for 1 h. After washing, erythrocytes at 1% hematocrit were incubated with transfected HEK293T cells at 37 °C for 2 h. After washing with PBS three times to remove unbound erythrocytes, the cells were visualized using Nikon Eclipse Ti-U inverted microscope (Nikon Corporation). The number of rosettes (more than 50% of the single HEK293T cell surface were covered by erythrocytes) (31) was counted in 10 fields at 200 × magnification under bright field microscopy. The transfection efficiency was evaluated by the proportion of GFP-positive cells in five fields at 200 × magnification under fluorescence microscopy. Images were captured by NIS-Elements software (Nikon Corporation) and manipulated by Adobe Photoshop CC.

### *P. falciparum* culture

*P. falciparum* 3D7 parasites were cultured on O^+^ human erythrocytes (4% hematocrit) in complete RPMI 1640 medium containing 10.44 g/L RPMI medium 1640 powder (Gibco), 5.96 g/L Hepes (Sigma), 2 g/L NaHCO_3_, 5 g/L Albumax (Sigma), 50 mg/L hypoxanthine II (Sigma), and 40 mg/L gentamicin in a 37 °C humidified incubator maintained with a gas mixture of 5% CO_2_, 5% O_2_, and 90% N_2_ according to Trager and Jensen (63).

### Invasion inhibition assays

To evaluate the effect of mouse anti-PfGAMA-Tr3 antisera, rabbit anti-ANK1-F2 antisera, and rabbit anti-band 3-P5 antisera on *P. falciparum* invasion, invasion inhibition assays were performed. Synchronized parasites at the late trophozoite or early schizont stage at 1.5% parasitemia were seeded into 96-well plates at 2.5% hematocrit in a final volume of 100 μl containing antisera. Anti-PfGAMA-Tr3 antisera, anti-ANK1-F2 antisera, and anti-band 3-P5 antisera at 1:10 dilution were added in the plates in triplicate and incubated with the parasite cultures for 18 h. Heparin served as an invasion inhibition positive control, while both mouse and rabbit PI sera at 1:10 dilution were included as negative controls. After incubation, the pellet was fixed with 0.05% glutaraldehyde and stained with SYBR Green I nucleic acid gel stain (1:5,000, Invitrogen) at 37 °C in the dark for 30 min. The postinvasion rate was quantified using an Accuri C6 plus flow cytometer (BD Biosciences) with 100,000 events per sample.

### Indirect immunofluorescence assay

Thin blood smears of *P. falciparum* 3D7 culture were air-dried, fixed with 4% paraformaldehyde for 20 min, and permeabilized in 0.1% Triton X-100 for 10 min. After blocking with 5% skimmed milk in PBS overnight at 4 °C, the slides were washed briefly with PBST and incubated with mouse anti-PfGAMA-Tr3 (1:50 dilution) and rabbit anti-ANK1-F2 (1:50 dilution) for 1.5 h at 37 °C. The slides were briefly washed and incubated for 30 min at 37 °C with the following secondary antibodies: Alexa-488 goat anti-mouse IgG (1:1,000, Invitrogen), Alexa-568 donkey anti-rabbit IgG (1:500, Invitrogen). After washing, coverslips were mounted using antifade mounting medium with DAPI (Vector Laboratories). Images were captured using a Zeiss LSM880 confocal microscope (Zeiss) and processed by Zen software (Zeiss).

### Statistical analysis

GraphPad Prism (GraphPad Software) was used to analyze all data. Student's *t* test was performed for the erythrocyte binding inhibition assay. One-way analysis of variance with Tukey's post hoc test was performed for the invasion inhibition assay.

## Data availability

All data used for the study are presented or cited in the main manuscript or the supporting information. The raw mass spectrometry data are available at Figshare (https://doi.org/10.6084/m9.figshare.17209637.v1).

## Supporting information

This article contains supporting information.

## Conflict of interest

The authors declare that they have no conflicts of interest with the contents of this article.

## Figures and Tables

**Figure:**
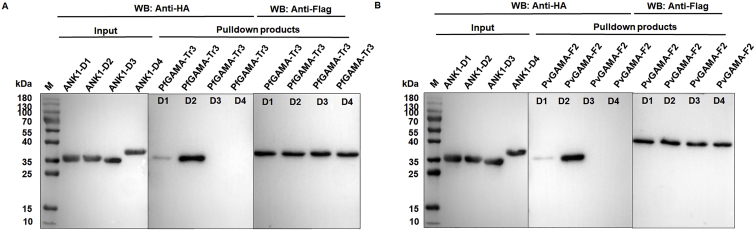
Figure S1**Interaction of GAMA with ANK1.***A* and *B*, Flag-pulldown assays showing that PfGAMA-Tr3 (*A*) and PvGAMA-F2 (*B*) bound to ANK1-D2 and weakly bound to ANK1-D1, but not to ANK1-D3, D4. HA-ANK1-D1, D2, D3, and D4 were incubated with immobilized Flag-PfGAMA-Tr3 or PvGAMA-F2, respectively. After washing the anti-Flag beads, the bound proteins were eluted and subjected to Western blot analysis using the indicated antibodies. ANK1, ankyrin 1; PfGAMA, *P. falciparum* glycosylphosphatidylinositol-anchored micronemal antigen.

**Figure:**
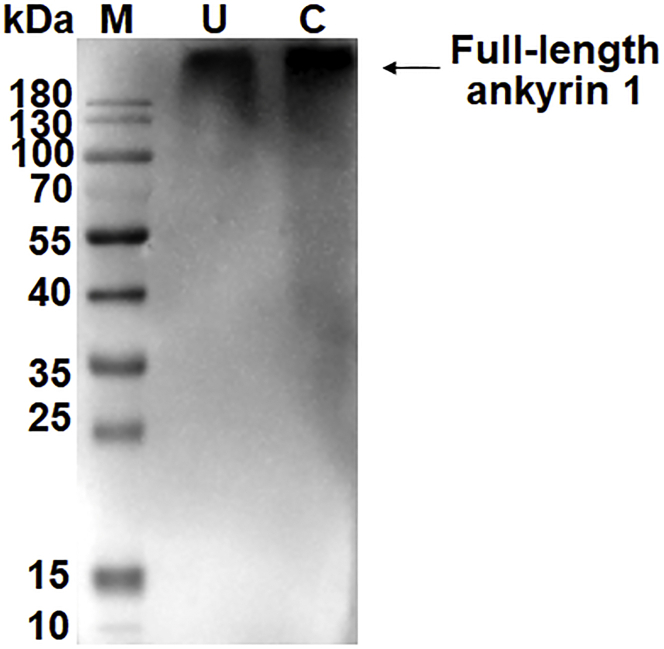
Figure S2**Chymotrypsin treatment hardly affected erythrocyte ANK1.** Western blot of solubilized untreated (U) and chymotrypsin-treated (C) erythrocyte ghosts. Rabbit anti-ANK1 antisera were used to detect erythrocyte ANK1. ANK1, ankyrin 1.

**Figure:**
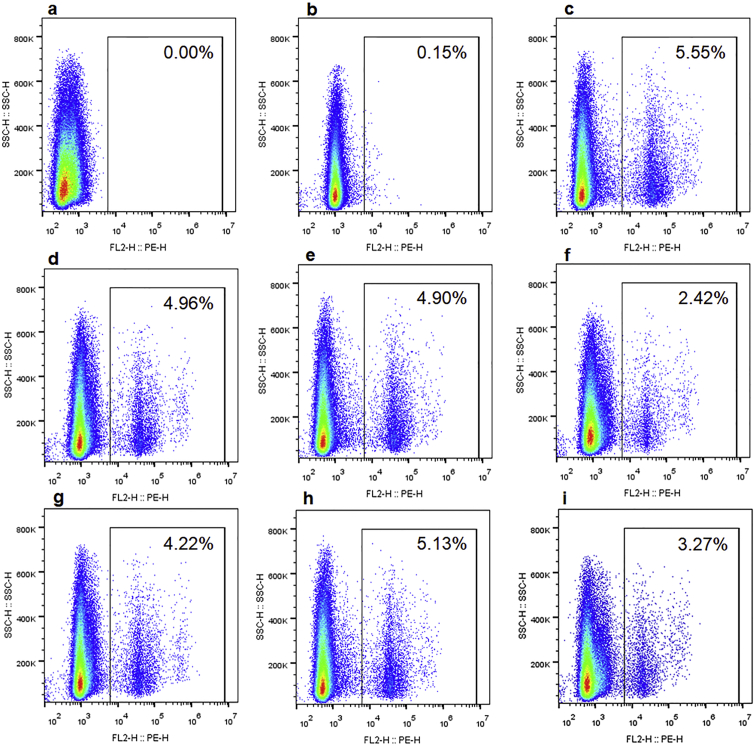
Figure S3**Representative flow cytometry scatter plots showing infection rates.** Panel *A*, unstained erythrocytes; panel *B*, uninfected erythrocytes; panel *C*, infected erythrocytes; panel *D*–*I*, infected erythrocytes treated with rabbit PI sera (*D*), mouse PI sera (*E*), heparin (*F*), mouse anti-PfGAMA-Tr3 sera (*G*), rabbit anti-ANK1-F2 sera (*H*) and rabbit anti-band 3-P5 sera (*I*).

**Figure:**
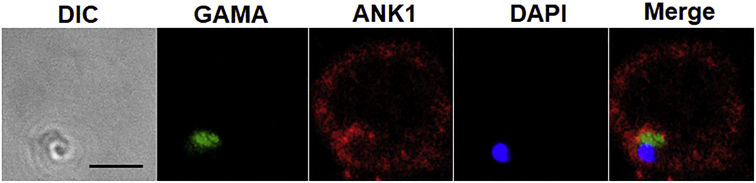
Figure S4**The close apposition of the plasma membrane of the newly invaded parasite and the erythrocyte membrane observed by IFA.***P. falciparum* parasites were probed with mouse anti-PfGAMA-Tr3 antisera followed by Alexa-488 goat anti-mouse IgG (*green*), and infected erythrocytes were probed with rabbit anti-ANK1-F2 antisera followed by Alexa-568 donkey anti-rabbit IgG (*red*). Parasite nuclei were stained with DAPI (*blue*). Scale bar, 3 μm. PfGAMA, *P. falciparum* glycosylphosphatidylinositol-anchored micronemal antigen; ANK1, ankyrin 1; IFA, indirect immunofluorescence assay.
