# Supplementary material for: Glycosylphosphatidylinositol-anchored micronemal antigen (GAMA) interacts with the band 3 receptor to promote erythrocyte invasion by malaria parasites
Source: J Biol Chem. 2022 Feb 21;298(4):101765. doi: 10.1016/j.jbc.2022.101765 (PMC8931436; doi:10.1016/j.jbc.2022.101765)
Supplement: Supplemental Tables S1–S5 [file mmc1.doc]

**Glycosylphosphatidylinositol-anchored micronemal antigen interacts with band 3****-ankyrin 1 complex promotes** **erythrocyte invasion by malaria parasites**

Jiachen Lu^1^, Ruilin Chu^1, 2^, Yi Yin^3^, Huijie Yu^1^, Qinwen Xu^1^, Bo Yang^1^, Yifan Sun^1^, Jing Song^4^, Qiubo Wang^5^, Jiahui Xu^3^, Feng Lu^3^ and Yang Cheng^1*^

**Tables S1 to S5 and Figures S1 to S4**

**Table S1. Identified peptide sequences of identified erythrocyte membrane proteins by LC-MS.**

| **No.** | **Protein** | **UniProt accession no.** | **No. of unique peptides** | **Peptide sequences** |
| --- | --- | --- | --- | --- |
| 1 | Ankyrin 1 | P16157 | 23 | AKDDQTPLHCAAR, CYCMTDDKVDK, DDQTPLHCAAR, DSGEGDTTSLR, EADAATSFLR, EIILETTTKK, ELVNYGANVNAQSQK, EPGGSLSFLR, GAQIETK, FLLQHQADVNAK, GASPNVSNVK, GEIVNMLEGSGR, PYSVGFR, HGVMVDATTR, ISEILLDHGAPIQAK, ITHSPTVSQVTER, LEGALSEEPR, LCQDYDTIGPEGGSLK, NGASPNEVSSDGTTPLAIAK, TPTPLALR, QNQVEVAR, TAAVLLQNDPNPDVLSK, VETPLHMAAR |
| 2 | Anion transport protein (band 3) | P02730 | 15 | AAATLMSER, ASTPGAAAQIQEVK, IDAYMAQSR, IPPDSEATLVLVGR, NVELQCLDADDAK, SVTHANALTVMGK, YQSSPAKPDSSFYK, ILLLFKPPK, VLLPLIFR, YHPDVPYVK, ADFLEQPVLGFVR, LSVPDGFK, FIFEDQIRPQDR, LQEAAELEAVELPVPIR, GWVIHPLGLR |
| 3 | α-Spectrin | P02549 | 7 | AAVGQEEIQLR, EQELQKEEAR, ESLNEAQK, MQHNLEQQIQAK, SYEDPTNIQGK, VLETAEEIQER, VLQEESQNK |
| 4 | protein 4.2 | P16452 | 6 | CCEDGTLELTDSNTK, CEDITQNYK, CLGIPAR, GVGSDRCEDITQNYK, NVELQCLDADDAK, SCDFQAAR |
| 5 | Protein 4.1 | P11171 | 6 | DVPIVHTETK, LAPNQTK, LTSTDTIPK, SLDGAAAVDSADR, SQEEIKK, TQTVTISDNANAVK |
| 6 | β-Spectrin | P11277 | 4 | GYQPCDPQVIQDR, LQTAYAGEK, LVSDINR, VDNVNAFIER |
| 7 | 55 kDa erythrocyte membrane protein | Q00013 | 1 | NALLSQNPEK |

**Table S2. Codon-optimized sequences used in this study.**

| Name | Codon-optimized sequences |
| --- | --- |
| *pfgama-tr3* | AAAGATATTATTAAACTGCTGAAAGATCTGATTAAATATCTGCACATTGTGAAATTTGAAAATAACGAACCGACCACCAACATTGACGAGGAAGGCATCCGCAAGCTGCTGGAGAACAGCTTCTTCGATTTAAACGACGACATTTTAATTGTTCGTTTACTGCTGAAACCGCAGACCGTGATTTTAACCGTGATCCAGAGCTTTATGCTGATGACCCCGAGTCCGAGCCGTGATGCCAAGGCCTATTGCAAGAAAGCTTTAATCAACGACCAGCTGGTGCCGACCAACGACACCAATATTTTAAGCGAGGAAAACGAGCTGGTGAACAACTTTAGTACAAAATATGTTCTGATCTATGAGAAAATGAAGCTGCAAGAACTGAAGGAGATGGAGGAGAGCAAACTGAAAATGAAATATAGCAAAACCAATTTAAGCGCACTGCAAGTTACCAACCCGCAAAACAACAAAGATAAAAATGATGCCAGCAATAAAAATAATAACCCGAATAATAGCAGCACCCCGCTGATTGCAGTGGTGACCGATCTGAGCGGCGAAAAGACCGAGGATATCATCAATAATAACGTGGATATTGCAACTTTAAGCGTGGGCGTGCAGAATACCTTTCAAGGTCCGAACGCCAAAGCC |
| *pvgama-f2* | GATGTTAGCGTGGATGAAAAAGGCGATCGCGCCACAACCGCTGGTGGCAACCAGAGCGCAAGCGTTGCCGCAGCAGCACCGAAAGATGCCGGTCCGACCGTTGCCGCACCGAATACCGCCGCAACACTGAAAACCGCAGCCAGTCCGAATGCCGCAGCAACCAATACCGCAGCACCGCCGAATATGGGCGCCACCAGCCCTCTGAGCAATCCGCTGTATGGTACCAGCTCTTTACAGCCGAAAGATGTGGCAGTTCTGGTGCGCGATCTGCTGAAAAACACCAACATCATCAAATTCGAGAACAACGAGCCGACCAGCCAGATGGACGATGAAGAAATTAAAAAACTGATTGAAAGCAGCTTCTTTGATTTAAGCGACAACACCATGCTGATGCGTTTACTGATTAAACCTCAAGCTGCCATTTTACTGATTATCGAAAGCTTTATTATGATGACCCCGAGCCCGACCCGCGATGCCAAAACCTACTGCAAAAAGGCTTTAGTGAACGGCCAGCTGATTGAAACCAGCGATTTAAACGCCGCCACAGAAGAGGATGATCTGATTAATGAATTTAGTAGCCGTTACAATTTATTTTACGAGCGTTTAAAGCTGGAAGAACTGCGCGAAATCGAGCAGAACCGTAAGGCTTTAAAAAACAGCAAGGGCACTTTAAGCGTGCTGGAGGTTGCAAACAGCCAGAATGCCCCGGATGGTAAGGGCGTTAATGGTAGCGGT |

**Table S3. Primer sequences of *ANK1* and *SLC4A1 (Band 3)* for recombinant protein expression and primer sequences of *pfgama-tr3* and *pvgama-f2* genes for HEK293T cell surface expression.**

| Primer | Sequences (5'→3')^a^ |
| --- | --- |
| *pfgama-tr3*-F | ggtcctggacGAATTCAAAGATATTATTAAACTGCTGAAAG |
| *pfgama-tr3*-R | gtgtatggggccttGGGCCCCTTATCGTCGTCATCCTTGTAATCGGCTTTGGCGTTCGG |
| *pvgama-f2*-F | ggtcctggacGAATTCGATGTTAGCGTGGATGAAAA |
| *pvgama-f2*-R | gtgtatggggccttGGGCCCCTTATCGTCGTCATCCTTGTAATCACCGCTACCATTAACGCC |
| *ANK1-F1*-F | gctgatatcGGATCCATGCCCTATTCTGTGGGC |
| *ANK1-F1*-R | gtggtggtgCTCGAGAGCGTAATCTGGAACATCGTATGGGTAGAGGTGGTCTCCCTGAGC |
| *ANK1-F2*-F | gctgatatcGGATCCGGCAACGTGATCATGGTG |
| *ANK1-F2*-R | gtggtggtgCTCGAGAGCGTAATCTGGAACATCGTATGGGTATTTTCCGGCAGCATTC |
| *ANK1-F3*-F | gctgatatcGGATCCACCACCGCCGGGC |
| *ANK1-F3*-R | gtggtggtgCTCGAGAGCGTAATCTGGAACATCGTATGGGTAGAAGCTGATGAGTTCTTCCC |
| *SLC4A1-L4*-F | gggcccctgGGATCCTTCATTCAGGATACCTACACCC |
| *SLC4A1-L4*-R | atgcggccgCTCGAGAGCGTAATCTGGAACATCGTATGGGTACATCCAGATGGGAAACTCG |
| *SLC4A1-L5*-F | gggcccctgGGATCCCTCAGTGCCACCACCGT |
| *SLC4A1-L5*-R | atgcggccgCTCGAGAGCGTAATCTGGAACATCGTATGGGTAGCGGGACAGGATGGG |
| *SLC4A1-L6*-F | gggcccctgGGATCCGACCGCATCTTGCTTCTG |
| *SLC4A1-L6*-R | atgcggccgCTCGAGAGCGTAATCTGGAACATCGTATGGGTACAGGGAGGCCGGC |
| *ANK1-D1*-F | gctgatatcGGATCCATGCCCTATTCTGTGGGC |
| *ANK1-D1*-R | gtggtggtgCTCGAGAGCGTAATCTGGAACATCGTATGGGTACTTGCTAAGAACGTCTGGGT |
| *ANK1-D2*-F | gctgatatcGGATCCACGGGATTCACGCCC |
| *ANK1-D2*-R | gtggtggtgCTCGAGAGCGTAATCTGGAACATCGTATGGGTACTCGGTGACCGCGTC |
| *ANK1-D3*-F | ctgatatcGGATCCTCTGGCCTGACACCTCTC |
| *ANK1-D3*-R | gtggtggtgCTCGAGAGCGTAATCTGGAACATCGTATGGGTACCAGGCAGGGCTGTG |
| *ANK1-D4*-F | ctgatatcGGATCCAATGGCTACACCCCTTTG |
| *ANK1-D4*-R | gtggtggtgCTCGAGAGCGTAATCTGGAACATCGTATGGGTAGAAGCTGATGAGTTCTTCCC |

^a^The vector sequences are lowercase, and the restriction sites are underlined. FLAG tag sequences and HA tag sequences are highlighted in gray.

**Table S4. Amino acid sequence of synthetic peptides used in this study.**

| Peptide name | Amino acid sequence |
| --- | --- |
| Band 3-P5 | LSATTVRSVTHANALTVMGKASTPGAAAQIQEVKEQRISGLLVAVLVGLSILMEPILSR |

**Table S5. Amino acid sequences of band 3 fragments expressed in this study.**

| Fragment | Amino acid sequences |
| --- | --- |
| L4 | FIQDTYTQKLSVPDGFKVSNSSARGWVIHPLGLRSEFPIWM |
| L5 | SATTVRSVTHANALTVMGKASTPGAAAQIQEVKEQRISGLLVAVLVGLSILMEPILSR |
| L6 | DRILLLFKPPKYHPDVPYVKRVKTWRMHLFTGIQIICLAVLWVVKSTPASL |
